# Digital Phenotyping for Mood Disorders: Methodology-Oriented Pilot Feasibility Study

**DOI:** 10.2196/47006

**Published:** 2023-12-29

**Authors:** Scott Breitinger, Manuel Gardea-Resendez, Carsten Langholm, Ashley Xiong, Joseph Laivell, Cynthia Stoppel, Laura Harper, Rama Volety, Alex Walker, Ryan D'Mello, Andrew Jin Soo Byun, Peter Zandi, Fernando S Goes, Mark Frye, John Torous

**Affiliations:** 1 Department of Psychiatry and Psychology Mayo Clinic Rochester, MN United States; 2 Beth Israel Deaconess Medical Center Boston, MA United States; 3 Research Application Solutions Unit Mayo Clinic Rochester, MN United States; 4 Johns Hopkins University Baltimore, MD United States

**Keywords:** mood disorders, depression, bipolar disorder, digital health, digital phenotyping, mobile apps, patient-generated health data, wearable devices

## Abstract

**Background:**

In the burgeoning area of clinical digital phenotyping research, there is a dearth of literature that details methodology, including the key challenges and dilemmas in developing and implementing a successful architecture for technological infrastructure, patient engagement, longitudinal study participation, and successful reporting and analysis of diverse passive and active digital data streams.

**Objective:**

This article provides a narrative rationale for our study design in the context of the current evidence base and best practices, with an emphasis on our initial lessons learned from the implementation challenges and successes of this digital phenotyping study.

**Methods:**

We describe the design and implementation approach for a digital phenotyping pilot feasibility study with attention to synthesizing key literature and the reasoning for pragmatic adaptations in implementing a multisite study encompassing distinct geographic and population settings. This methodology was used to recruit patients as study participants with a clinician-validated diagnostic history of unipolar depression, bipolar I disorder, or bipolar II disorder, or healthy controls in 2 geographically distinct health care systems for a longitudinal digital phenotyping study of mood disorders.

**Results:**

We describe the feasibility of a multisite digital phenotyping pilot study for patients with mood disorders in terms of passively and actively collected phenotyping data quality and enrollment of patients. Overall data quality (assessed as the amount of sensor data obtained vs expected) was high compared to that in related studies. Results were reported on the relevant demographic features of study participants, revealing recruitment properties of age (mean subgroup age ranged from 31 years in the healthy control subgroup to 38 years in the bipolar I disorder subgroup), sex (predominance of female participants, with 7/11, 64% females in the bipolar II disorder subgroup), and smartphone operating system (iOS vs Android; iOS ranged from 7/11, 64% in the bipolar II disorder subgroup to 29/32, 91% in the healthy control subgroup). We also described implementation considerations around digital phenotyping research for mood disorders and other psychiatric conditions.

**Conclusions:**

Digital phenotyping in affective disorders is feasible on both Android and iOS smartphones, and the resulting data quality using an open-source platform is higher than that in comparable studies. While the digital phenotyping data quality was independent of gender and race, the reported demographic features of study participants revealed important information on possible selection biases that may result from naturalistic research in this domain. We believe that the methodology described will be readily reproducible and generalizable to other study settings and patient populations given our data on deployment at 2 unique sites.

## Introduction

Over the past decade, the rapidly developing field of mental health research has focused on leveraging digital hardware like smartphones and wearables with their ever expanding and refining arrays of sensors and software innovations to facilitate user data collection [1]. The general goal of this area of mental health research is to gain novel insights into at least two central domains: predicting changes in individual mental health status and defining how mental illness may mediate use patterns of digital interfaces [2-4]. Digital phenotyping is an evolving research field [1], and in the setting of different contexts and connotations to the term digital phenotyping, we will turn to the following definition of digital phenotyping as provided by Dr Thomas Insel in a paper widely recognized as an impetus for accelerating interest in this research field: “the moment-by-moment quantification of the individual-level human phenotype in situ using data from personal digital devices, in particular smartphones” [5].

At this initial exploratory phase of digital phenotyping, there is limited understanding of what specific patient data streams and analyses may be most useful in interpreting illness and providing insights with diagnosis clarification, treatment recommendations, and more precise prognostic predictions [6]. The promise of digital phenotyping for mental illness is predicated on a wide range of biological, psychological, and social factors that impact the presentation of most, if not all, psychiatric illnesses. However, the question of how to most effectively collect wide-ranging biopsychosocial data remains an active area of investigation. Research in this domain must provide insights on how best to evolve the dimensionality (ie, the number of variables and frequency of data collected) of patient data streams, the forms of data collected (ie, the nature of the variables themselves), and the higher-order patient features for which these variables serve as proxy metrics (ie, the biopsychosocial feature that a metric is intended to track), as well as the mechanism of data collection (ie, active or passive collection from the patient).

In relation, this research needs to be understood in the context of any potential biases. While there have been prior studies using digital phenotyping methods for mood and bipolar disorder [7,8], the focus has often been the resulting behavior features and not an assessment of which types of people agree to partake in this research and the quality of the data gathered from their phones. Both factors matter in the interpretation of later results. For example, a recent large-scale digital phenotyping study of people with depression noted that data quality precluded any discussion of the clinical meaning of the signal data [9], and a recent review of digital phenotyping for depression noted that high heterogeneity in the results is likely due in part to a lack of reporting on underlying data quality [9].

In this article, we describe several components of study methodology that facilitate reproducibility, with a focus on patient interest and acceptability. First, we describe the protocol design for structuring a 12-week case-control pilot feasibility study in treatment-seeking depressed patients. Second, we provide initial insights from the initial implementation of the protocol to describe the barriers and challenges we encountered for successful recruitment, longitudinal study participation, and data collection. Additionally, we assess the acceptability of passive data collection with a smartphone in depressed patients and investigate how passive data gathered via technology platforms may subsequently be analyzed to generate transdiagnostic digital phenotypes that can potentially inform the assessment and treatment outcomes of major mood disorders.

## Methods

### Study Design and Patient Recruitment

#### Study Population

This is a multisite study that involved recruitment at Mayo Clinic (Rochester, Minnesota) and Johns Hopkins University (Baltimore, Maryland).

Adult patients (18 to 65 years of age) seeking treatment for major depressive disorder (MDD; sample size=49) or bipolar disorder (sample size=28; 13 with type I bipolar disorder and 15 with type II bipolar disorder) with no current active comorbid psychiatric disorder, suicidal ideation, or psychosis were eligible to participate in this study. Controls (aimed sample size=31) were defined as individuals with no active psychiatric disorder in the previous year. A history of psychiatric disorders was not considered an exclusion criterion.

This study focused on a patient population with a previously diagnosed mood disorder for several reasons. There are parallel studies by several members of this research group, and the co-authors on this study have ongoing research and recent published studies involving the MindLAMP app for digital phenotyping investigations that focus on other patient populations, namely those of psychotic disorders [10] and substance use disorders [11]. The existing literature on digital phenotyping of mood disorders is limited and warrants its own dedicated investigation. Collectively, we seek to evaluate the feasibility of studying different psychiatric populations in pursuit of insights derived from digital phenotyping.

We grouped patients according to the Diagnostic and Statistical Manual of Mental Disorders, Fifth Edition (DSM-V) diagnosis, with the goal of assessing whether data quality and demographic differences were appreciated between groups. The naturalistic study recruitment approach in this pilot study across these diagnostic categories was intended to provide information on potential biases in recruitment and, in turn, inform efforts toward awareness and correction of intergroup differences if needed to optimize a future study. Our recruitment also attempted to implement a naturalistic approach that would provide insights about potential data quality differences that may be related to diagnostic differences, symptom burden, or demographic features of participants.

The study participants received US $25 for their time during each visit (baseline, month 1, month 2, and month 3; a total of US $100), but not for engagement with the app. Participants who borrowed a smartwatch from the study team were offered the watch for free upon study completion (an approximately US $40 value). As the app does not use any smartphone data because such data are transferred only via Wi-Fi, there were no data costs to cover.

#### Assessment Measures

**Figure 1:**
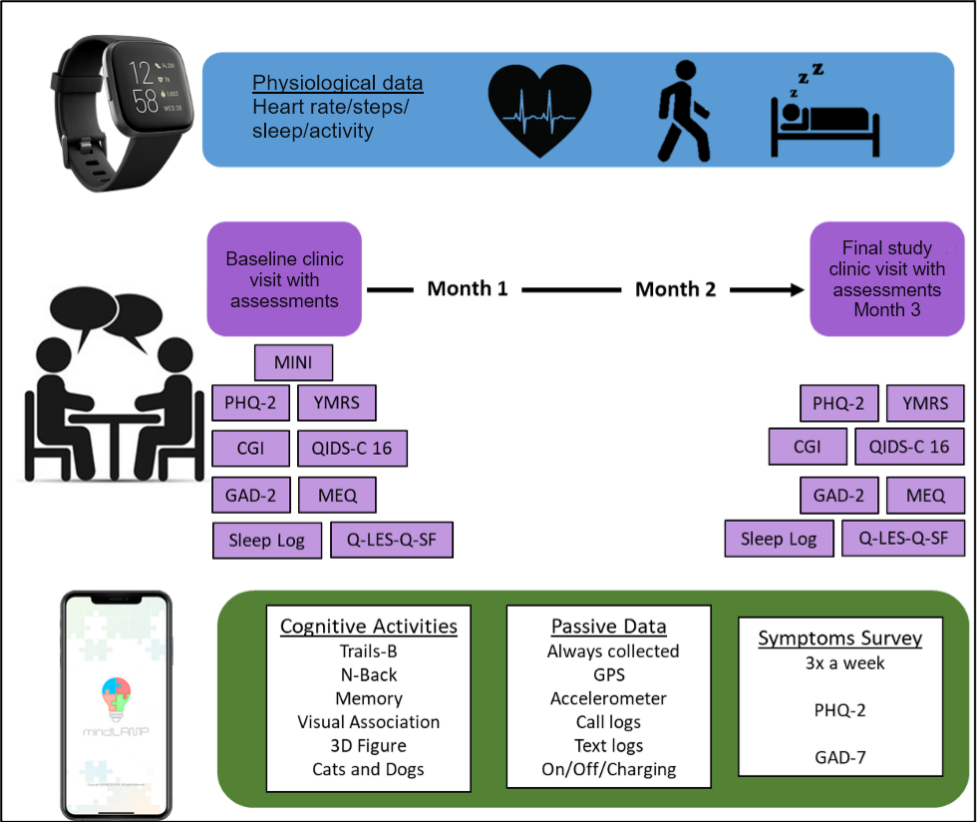
Schematic diagram of the study design and the passively and actively collected data. CGI: Clinical Global Impression; GAD-2: Generalized Anxiety Disorder-2; MEQ: Morningness-Eveningness Questionnaire; MINI: Mini International Neuropsychiatric Interview; PHQ-2: Patient Health Questionnaire-2; QIDS: Quick Inventory of Depressive Symptomatology; Q-LES-Q-SF: Quality of Life Enjoyment and Satisfaction Questionnaire – Short Form; YMRS: Young Mania Rating Scale.

Diagnostic confirmation was obtained through the Mini International Neuropsychiatric Interview (MINI), a valid instrument for assessing depression in primary care settings [12,13]. The severity of depressive symptoms was assessed at baseline and follow-up visits using the Quick Inventory of Depressive Symptomatology – Clinician Rating (QIDS), which was considered the primary outcome measure of baseline to endpoint change [14]. Hypomanic/manic symptoms were ruled out during each in-person visit using the Young Mania Rating Scale (YMRS) [15]. Six raters (2 psychiatrists and 4 clinical research staff) participated in the rating of video-taped interviews using the QIDS and YMRS to quantify the agreement among study team members. Interrater reliability coefficients were 0.92 for the QIDS and 0.96 for the YMRS, indicating an elevated level of agreement [16]. Participants’ circadian rhythm phenotypes were assessed with the Morningness-Eveningness Questionnaire (MEQ) at the first and final visits [17]. During visits, participants were asked to complete a self-report measure of enjoyment and satisfaction in multiple areas of daily functioning (Quality of Life Enjoyment and Satisfaction Questionnaire–Short Form [Q-LES-Q-SF]) [18]. Finally, participants were asked to keep a sleep log the week prior to each visit, based on the American Academy of Sleep Medicine’s sleep diary. A complete list of measures of illness severity and clinical phenotyping is shown in Figure 1.

The study required use of a compatible smartphone device (ie, Android or iOS device) able to run the study app, as well as a willingness to wear a smartwatch with an accelerometer or Oura Ring with congruent functionality. Participants who owned a smartwatch were asked to allow research access and use of the passive data collected. Participants who did not own a smartwatch were provided with a smartwatch (Willful smartwatch [Willful Technology Co, Ltd] or LETSCOM ID205L smartwatch [Lets Fit]) or an Oura Ring to be used throughout the study period. Participants were informed that the device must be in proximity (approximately 100 feet) to the smartphone to enable data to be collected. Of note, all patients who enrolled in the study using their own wearable device had an Apple Watch, though other wearable devices would have been permitted as alternatives if patients with such self-owned devices would have participated. The exclusivity of the Apple Watch was incidental.

#### Safety Monitoring

Throughout the study, participants adhered to their routine clinical care for depression and were educated about the fact that the collected data were not monitored in real-time. They were also shown how the app can enable them to reach out for help at any time (ie, calling 911 and connecting to a primary care provider).

### Ethical Considerations

The protocol was approved by the institutional review board at each study site (Mayo Clinic: 20-008865; Johns Hopkins University: IRB00285631), and every participant was required to provide written informed consent to participate in this study.

### Informatics Architecture

Digital phenotyping is predicated on simultaneously collecting high-dimensional sets of variables with massive data streams for many if not all variables sampled. The structures of data ingestion, database maintenance, and security and accessibility of research data require prospective planning and development prior to research implementation.

#### MindLAMP App

MindLAMP is an open-source smartphone app developed for research and clinical purposes by the Digital Psychiatry Division at Beth Israel Deaconess Medical Center, a Harvard Medical School teaching hospital. The app collects 3 types of data: active, passive, and meta-data. Active data refer to data from surveys or cognitive assessments that require the user to actively engage for data to be collected. Examples include offering the Patient Health Questionnaire-9 (PHQ-9) or Generalized Anxiety Disorder-7 (GAD-7) scales on the app. MindLAMP provides user functionality to input any clinical screening tool and adjusts the frequency with which it is pushed to users. Passive data include, for example, GPS, accelerometer, gyroscope, and screen state data, which are captured directly from the phone without any active user engagement. Researchers can harness analysis pipelines, such as LAMP Cortex [19,20], to transform this raw data into clinically meaningful features (eg, home time), which can subsequently be used for more advanced tasks. Meta-data refer to information about how the app is used and can include time to respond to each survey question (latency) and information about when the app is used and for how long.

#### Data Collection, Storage, and Security

**Figure 2:**
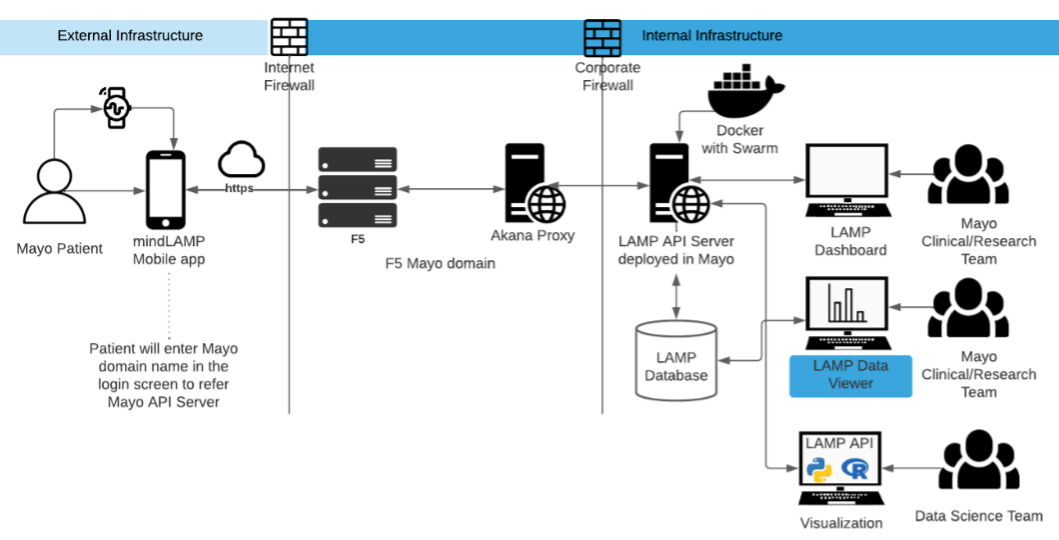
Diagram of the MindLAMP backend architecture. API: application programming interface.

The MindLAMP app collected various passive and active data from the smartphones and smart watches used by the study patients. These data included activity levels; credentials; and participant, researcher, study, and sensor data. MindLAMP stored these data in CouchDB and MongoDB formats, which involve a NoSQL database. At Mayo Clinic, the VeryFitPro wearable and companion smartphone app were used to collect additional sensor data. The data from the smartwatch was integrated into either Apple Health Kit or Google Fit (depending on the type of phone used) and then sent to the MindLAMP app. Data from the phone were automatically transferred using the phone’s application programming interface (API) in JavaScript object notation (JSON) format, which was then captured and sent to various databases by the application source code (see Figure 2).

The MindLAMP app consists of 2 parts: (1) a main dashboard developed by Beth Israel Deaconness Medical Center, which provides study, participant, questionnaire, resource management, and Cortex modules for visualization, and (2) a visualization dashboard developed by Mayo Clinic with views for all activity levels collected (ie, step count, heart rate, sleep data, telephony, screen state, GPS data, accelerometer data, and analytics), and GAD-7 and PHQ-9 scores. Summary views were available at both patient and study levels (see Figure 2). For all GPS data collected, all analyses detected changes from a personal baseline of mobility recorded by GPS. These data can be deidentified by deriving a metric of entropy (eg, time and distance away from the GPS basepoint) through automated calculations so that identifiable personal location information is not accessible to researchers as a metric.

### Vectors of Digital Phenotyping Data

#### Active Data

Smartphone surveys have been previously used to assess clinical symptoms in bipolar I disorder and schizophrenia, identifying a substantial convergence between collected data in the patient’s naturalistic environment and clinic-based assessments of these disorders, and suggesting the reliability of digital tools used to measure mood [21,22]. In our study, clinical data were collected from answers to the prompted ecological momentary assessments (EMAs) that appeared in the MindLAMP app 3 times a week. These surveys included a brief screen for depression, the Patient Health Questionnaire-2 (PHQ-2), and the Generalized Anxiety Disorder-2 (GAD-2) for anxiety. The related questionnaires PHQ-9 and GAD-7 were initially planned as study EMAs, but the ease of completion for patients and comparable validity resulted in switching to the more abbreviated versions. Of note, all individuals enrolled in the study were also in routine clinical care and were thus receiving clinical safety assessments. Additional baseline and study completion surveys were collected to gather more comprehensive psychological profile information about the patient’s symptoms and to measure changes that may have occurred during the study enrollment period.

#### Passive Data

Perhaps the most important aspect of leveraging digital phenotyping is the collection of diverse streams of patient data across many domains of activity in an entirely passive manner [23].

##### Physiologic Metrics

Physiologic measures are a key area of innovation with the rapidly expanding array of sensors embedded in smartphones and consumer wearable devices [23-25]. Our study, which focused on the feasibility of tracking physiologic metrics, included step count, heart rate, and sleep quantification.

##### Social Metrics

Social dynamics are an important aspect of general individual wellness and can be a specific aspect of monitoring patient symptoms across a multitude of psychiatric illnesses, including bipolar disorder [26]. Among numerous possibilities, without collecting any private data about the communication itself, text message frequency, message length, latency between messages, call duration, and outgoing or incoming communication status all have potential value in describing the functional aspects of social interaction [27-29].

##### Geospatial Location

Physical mobility throughout a person’s geographic environment is another essential domain with wide ranging ability to describe behavioral patterns. GPS data provide an expansive set of raw data that can be used to derive more clinically relevant metrics, such as circadian movement (ie, the regularity of daily movement patterns), normalized entropy (ie, mobility between usual locations), and location variance (ie, quantity of physical movement irrespective of location), which have previously been shown to be statistically significant correlates of changes in reported mood patterns [28]. Our study collected GPS data, with the potential to leverage this to conduct further analyses of circadian movement, normalized entropy, and location variance, among other possible novel inquiries.

##### Metadata

Metadata, which have been described as passively collected data related to how a user interacts with the user interface of a specific app or operating system (eg, click time in responding to a survey question), can be considered a novel form of data having potential clinical significance and less ethical concern than more personally sensitive information like geospatial location or speech profile [29,30]. Metadata contain less identifiable information, and recent research has shown that comfort in sharing digital phenotyping data depends on the “level of detail of the data sensor” [31].

#### Poststudy Completion Participant Data Quality

In this interim analysis, we excluded study participants from the analysis if they did not actively complete baseline symptom tracking surveys. During the study, we switched from the PHQ-9 and GAD-7 EMA scales to the PHQ-2 and GAD-2 owing to interim formal feedback from study participants suggesting that participation drop off may be at least partially related to survey fatigue. While we did not track and evaluate this feedback quantitatively, we attempted to use appropriate judgement to optimize study engagement. In the setting of some initial participant feedback suggesting survey fatigue, we switched to the abbreviated PHQ-2 and GAD-2 symptom tracking scales during the study recruitment and implementation phases.

## Results

### Barriers to Patient Engagement

#### Patient Self-sufficiency With Digital Engagement

An essential element of our study is the consideration of digital literacy and access to broadband connectivity [32,33]. As part of the screening process, patients’ internet access and digital literacy were assessed. In addition, once recruited and after providing consent, participants were provided with personal assistance by a study coordinator to download the app, set up an account, and obtain basic training to navigate the app. For passive data collection through wearable devices, no digital proficiency with smartwatches was needed for the study.

#### Engagement With EMAs

Inclusion of repeated real-time assessments of psychological-related variables (eg, PHQ-9 and GAD-7 tests for depressive and anxiety symptoms), known as EMAs, can offer insights into the day-to-day course of psychiatric symptoms and rates of adherence and habituation to the app [34].

#### Recruitment and Enrollment

Given the unique data capture properties of this study, it is important to understand the relative engagement of each patient to understand any potential bias in the reported data. Few studies report on measures of data quality (eg, proportion of anticipated data collected [actual data]/[expected data]), which can be useful for recruitment planning and sample size planning for related research.

#### Study Participant Demographic Features and Data Quality Analysis

On completion of the recruitment phase of this study, several basic demographic variables were reviewed and analyzed across the 2 study sites (Table 1).

**Table 1:** Demographic features of the study participants.

Variable			Overall (N=90)		BPI^a^ (n=10)		BPII^b^ (n=11)		MDD^c^ (n=37)		Control (n=32)
**Age (years), mean (SD)**											
	Group	34.5 (13.9)		36.7 (7.1)		38.8 (15.3)		35.3 (11.9)		31.5 (16.7)	
	Male	34.0 (15.5)		37.0 (18.4)		37.8 (9.6)		39.3 (14.9)		28.2 (17.5)	
	Female	34.9 (13.4)		36.6 (4.0)		39.4 (18.4)		34.7 (11.2)		32.9 (16.5)	
**Gender identity, n (%)**											
	Male	23 (26)		2 (20)		4 (36)		7 (19)		10 (31)	
	Female	66 (73)		8 (80)		7 (64)		29 (78)		22 (69)	
	Nonbinary	1 (1)		0 (0)		0 (0)		1 (3)		0 (0)	
**Racial identity, n (%)**											
	White	62 (69)		8 (80)		9 (82)		28 (76)		17 (53)	
	Black or African American	8 (9)		1 (10)		0 (0)		5 (14)		2 (6)	
	Asian	13 (14)		0 (0)		1 (9)		1 (3)		11 (34)	
	American Indian	1 (1)		0 (0)		1 (9)		0 (0)		0 (0)	
	Mixed race	4 (4)		1 (10)		0 (0)		1 (3)		2 (6)	
	Not disclosed	2 (2)		0 (0)		0 (0)		2 (5)		0 (0)	
**Ethnicity, n (%)**											
	Hispanic or Latino	4 (4)		0 (0)		0 (0)		1 (3)		3 (9)	
	Not Hispanic or Latino	86 (96)		10 (100)		11 (100)		36 (97)		29 (91)	
**Highest level of education, n (%)**											
	High school diploma/GED^d^ equivalent	6 (7)		2 (20)		2 (18)		0 (0)		2 (6)	
	Some college, no degree	21 (23)		4 (40)		4 (36)		10 (27)		3 (9)	
	2-year associate	4 (4)		0 (0)		0 (0)		2 (5)		2 (6)	
	4-year bachelor	35 (39)		2 (20)		3 (27)		12 (32)		18 (56)	
	Some graduate school, no degree	6 (7)		0 (0)		1 (9)		3 (8)		2 (6)	
	Graduate school degree	18 (20)		2 (20)		1 (9)		10 (27)		5 (16)	
**Operating system, n (%)**											
	iOS	76 (84)		8 (80)		7 (64)		32 (86)		29 (91)	
	Android	14 (16)		2 (20)		4 (36)		5 (14)		3 (9)	
**Wearable device, n (%)**											
	Apple Watch	55 (61)		6 (60)		7 (64)		23 (62)		19 (59)	
	Willful	28 (31)		4 (40)		3 (27)		10 (27)		11 (34)	
	Oura Ring	7 (8)		0 (0)		1 (9)		4 (11)		2 (6)	
**Baseline symptom burden score, mean (SD)**											
	PHQ-2^e^	1.63 (1.10)		2.37 (2.35)		2.24 (1.63)		2.21 (1.40)		0.10 (0.17)	
	GAD-2^f^	2.07 (1.29)		3.19 (2.04)		2.32 (1.73)		2.74 (1.50)		0.42 (0.66)	

^a^BPI: bipolar I disorder.

^b^BPII: bipolar II disorder.

^c^MDD: major depressive disorder.

^d^GED: General Educational Development.

^e^PHQ-2: Patient Health Questionnaire-2.

^f^GAD-2: Generalized Anxiety Disorder-2.

##### Age

Between the 2 study sites, differences in mean study participant age were not statistically significant. Additionally, there was no statistically significant difference in the mean age of study participants based on their diagnosis of unipolar depression (35.3 years old), bipolar I disorder (36.7 years old), or bipolar II disorder (38.8 years old), or their identification as healthy controls (31.5 years old).

##### Gender

The proportion of female to male participants demonstrated a predominance of female participation across every diagnostic subgroup of patients when both study sites were aggregated, with the subgroup nearest to parity being the bipolar II disorder patients (7/11, 64% females and 4/11, 36% males within the diagnostic group) and that with the highest discrepancy being the bipolar I disorder patients (8/10, 80% females and 2/10, 20% males within the diagnostic group).

##### Phone Type

There was a preponderance of iOS users who were in this study in each subgroup. The proportion of iOS users was the lowest in the bipolar II disorder subgroup (7/11, 64% iOS within the diagnostic group) and the highest in the healthy control subgroup (29/32, 91% iOS within the diagnostic group). The overall proportion of study participants using iOS was 84% (76/90). Patients were given a wearable device for passive data tracking if they did not enter the study with a wearable device compatible with the study parameters. The majority of participants used an Apple Watch (55/90, 61%), all of which were personal devices that the patient used, and the patient declined the offer of a free wearable provided through the study. Of the remainder, 31% (28/90) used an Android-compatible Willful smartwatch and 8% (7/90) used an Oura Ring. The Oura Rings were provided exclusively at the Johns Hopkins study site based on device procurement preferences, which differed by study site.

### Data Quality Analysis

Smartphone sensors are generally collected at a researcher-defined default frequency. While collecting passive smartphone data, however, software settings or low app engagement can decrease the true frequency of the data collected. To quantify the true frequency of the data collected, for each participant, we split all the passive data collected during the study period into hour-long bins, consistent with the methodology of binning common to other studies [35,36]. We defined per participant data quality as the percentage of these bins that contained at least one smartphone GPS data point for each participant. Although GPS was not the only source of passively collected data, GPS data quality served as a consistent indicator of data quality in general (ie, high GPS data quality is closely associated with high data quality overall). We analyzed data quality differences across study sites, race, age groups, self-identified sex, and baseline symptom severity. Of note, in analyzing passive data quality, we only included participants who completed symptom surveys in the analysis.

#### Study Site

We calculated mean data quality across participants from the Johns Hopkins University and Mayo Clinic sites. Johns Hopkins participants had a mean GPS data quality of 0.29 (SD 0.34), whereas Mayo Clinic participants had a mean data quality of 0.67 (SD 0.34). This difference was found to be significant (*P*<.001) by the *t*-test.

#### Race

Participants were broken down into self-identified race groups, and mean data quality was calculated. Participants who identified as white had a mean data quality of 0.48 (SD 0.38), participants who identified as black had a mean data quality of 0.49 (SD 0.40), and participants who identified as Asian had a mean data quality of 0.38 (SD 0.42). These differences were not found to be significant (*P*=.68) by ANOVA.

#### Age

We separated participants into age ranges and compared differences in mean data quality, combining participants from both sites. Participants under 35 years had a mean data quality of 0.38 (SD 0.39), participants older than 35 and up to 60 years had a mean data quality of 0.47 (SD 0.37), and participants older than 60 years had a mean data quality of 0.79 (SD 0.24). ANOVA showed that these differences were significant (*F*_2,94_=3.27; *P*=.04).

#### Sex

Mean data quality among those who identified as male was 0.41 (SD 0.39), and mean data quality among those who identified as female was 0.50 (SD 0.39). This difference was not found to be statistically significant (*P*=.31) by the *t*-test.

#### Diagnosis

We also analyzed data quality differences among participant diagnosis groups. Control participants had a mean data quality of 0.43 (SD 0.40), participants with MDD had a mean data quality of 0.47 (SD 0.41), and participants with bipolar disorder had a mean data quality of 0.37 (SD 0.34). ANOVA showed no significant difference in data quality between these groups (*P*=.65).

#### Symptom Severity

We analyzed data quality differences among symptom severity as measured according to the PHQ-2 and GAD-2 scores. Patients were grouped in tertiles, and comparisons within the depression scores and anxiety scores were made for differences in data quality. Within the GAD-2 tertiles, the low tertile had a mean data quality of 0.37 (SD 0.35), the middle tertile had a mean data quality of 0.53 (SD 0.40), and the high tertile had a mean data quality of 0.42 (SD 0.35). There was no statistically significant difference in data quality among the tertiles of GAD-2 scores (*P*=.92). Within the PHQ-2 tertiles, the low tertile had a mean data quality of 0.42 (SD 0.36), the middle tertile had a mean data quality of 0.43 (SD 0.36), and the high tertile had a mean data quality of 0.46 (SD 0.40). ANOVA showed that there was no statistically significant difference in data quality among the tertiles of PHQ-2 scores (*P*=.19).

## Discussion

Combining passive and active data collection can contribute to the conceptualization of digital phenotypes in mood disorders. Our study methodology yielded results that did not demonstrate a statistically significant difference in data quality between gender and race, though data quality increased with age. This could be indicative of better study compliance among older participants. This is important for framing future results and understanding the generalizability of digital phenotyping in mood disorders. Our results of data quality are higher than related studies and sufficient to construct behavioral features. This is notable because the app used in this study is open-source and used across a range of mental health studies (depression, anxiety, and schizophrenia), suggesting the potential of reusable and free software for this research without the need for customized and expensive approaches. This helps ensure that digital phenotyping research can become more accessible and reproducible. While we did observe a difference in enrollment based on gender, the result provides an actionable target for future studies and is also in line with enrollment in research studies, suggesting that there is no additive recognized bias for digital phenotyping work.

We believe that this study provides a reproducible and readily generalizable methodology for using actively and passively collected clinical data to gain novel phenotypic insights about mood disorders. Our study used the MindLAMP app that was designed and developed with close involvement of clinicians with psychiatric domain expertise, which we believe has been an underlying asset in promoting the clinical reliability of our data collection and future analyses [29]. Furthermore, we present a review of key evidence on psychiatric phenotyping-related research to identify evidence-based techniques and current best practices for research and implementation [7,22,30].

There are several components of this methodology, which enhance the acceptability and feasibility of our approach to other digital phenotyping research endeavors. To date, mobile health apps are not obligated to comply with existing Health Insurance Portability and Accountability Act (HIPAA) rules and regulations, which adds a layer of complexity in attempting to introduce innovative technology in clinical practice that might improve patient outcomes [37]. Our study design addresses data security concerns related to confidentiality and protection of health information with mental health apps by storing the collected data (restricted and encrypted) on secured servers within the institution, without linkage to participants’ clinical data. Additionally, at different time points during the study, usability and acceptability of the mobile app were assessed, ensuring that participants can provide their input through qualitative and quantitative surveys, which attempt to assess the app’s user interface/user experience. These data can be used for informed iterative program design improvement. Finally, the inclusion of a healthy control group allowed the possibility to identify potential digital biomarkers of depression. A recent review by De Angel et al showed that depressive symptoms were associated with lower daily step count and inconsistent reporting (ie, missing data) [38]. This suggests that in addition to specific behaviors seen in depression (eg, social isolation), different patterns of engagement with technology may be conceptualized as a phenotypic expression of depression. In this regard, our clinical design and statistical analysis plan presented here include several strengths that address the limitations reported in previous studies in digital psychiatry, such as opportunistic study designs, inconsistent reporting (ie, missing data), and lack of generalizability [38].

In this naturalistic recruitment process, there were some important insights related to the study participants’ demographic features. First, the mean participant age across subgroups remained in the range of 30 to 39 years, and the participants were predominantly female. We did not collect data on participant race/ethnicity, socioeconomic status, or educational background. Existing literature has begun to explore different phenotypic patterns of technology usage between and within various demographic groups. There is nearly unbounded opportunity for future research to explore patterns of technology use as it may relate to biological and cultural demographic features. For example, recent studies designed a statistical learning model that was able to establish phenotypic clusters of phone call behaviors along a morningness-eveningness spectrum in older adults and demonstrate the descriptive power of the circadian rhythms of these individuals [39,40]. We recognize that the study described here certainly has biases and limitations associated with naturalistic sampling. In turn, this study sample lacked sufficient statistical power to interrogate potential behavioral phenotypes that may vary by demography and cultural factors. We believe that this opportunity will be adequately addressed through the growing depth and breadth of digital phenotyping investigation in the field at large, which will attempt to probe specific demographic questions related to differences in digital device usage as a function of age, educational background, social roles (eg, single, married, parents, and employment), race, sexual and gender orientation, cultural background, comorbid medical conditions, and numerous other demographic and sociological features.

Additionally, this study showed that subjects had a much higher likelihood of using iOS devices than Android devices, which resulted in differences in both hardware and software interfaces among study participants. Given that there were so few Android phones compared with Apple phones, we did not assess these differences. Differences between operating platforms may impact data quality, but given that different analyses may require different quality of data (eg, hour-by-hour analysis versus weekly summaries), it is hard to set an absolute bar for data quality. We hypothesize that differences in operating systems may also have some predictive power in defining participants’ socioeconomic status, though this remains untested in this study and is subject to future investigation.

We anticipate some limitations of this study. Combining patients with MDD and bipolar depression provides more clinical diversity, and while these illnesses share common symptomatology, treatment strategies and the course of illness are different [41]. Our future research plans seek to analyze individual and group phenotypic differences with active disordered mood symptoms, which may help clarify phenotypes that are either internally consistent for any given individual (ie, change from the baseline state of an individual but not necessarily consistent between individuals) or generalizable to group consistency (ie, interpersonally consistent phenotypes of mood disorder states as grouped by DSM-V diagnosis or novel proposed clustering).

In future analyses of these data sets, we will use novel methods to combine multiple data streams (ie, mobile survey assessments, mobile cognitive assessments, and behavioral and social data) gathered through passive data streams. We will use generalized linear models to assess differences between depressed patients and matched controls for each outcome at both baseline and the end of the study: research participant satisfaction, intent to continue use, perceived increased value to everyday lifestyle, perceived increased value to depression care management, clinical staff assessment of procedural fit to clinical practice, and added value to clinical practice. In all the statistical models described, we will adjust for the matched variables of age and sex.

Recognizing the potential clinical value of passive monitoring of affective disorders and closer tracking of depressive symptoms, and standardizing how mobile health studies are structured and analyzed are necessary to advance this growing field [8,38]. Hence, in these preliminary findings, we provide a comprehensive description of the methodology of our study assessing the feasibility of using a mobile health app in depression, a description of the technology used for the study, and the factors and barriers that need to be considered in iteratively developing a future study design in this domain. This work also considers the possible challenges with selection biases that must be carefully navigated in digital phenotyping research. In the context of research in digital psychiatry, increasing the availability of technology platforms and well-structured and consistent methodologies can potentially enable more efficient study implementation and widespread deployment of effective mobile health interventions.

## Abbreviations

DSM-V: *Diagnostic and Statistical Manual of Mental Disorders, Fifth Edition*
EMA: ecological momentary assessment
GAD-2: Generalized Anxiety Disorder-2
GAD-7: Generalized Anxiety Disorder-7
MDD: major depressive disorder
PHQ-2: Patient Health Questionnaire-2
PHQ-9: Patient Health Questionnaire-9
QIDS: Quick Inventory of Depressive Symptomatology
YMRS: Young Mania Rating Scale
